# Assessing the Construct Validity and Internal Reliability of the Screening Tool Test Your Memory in Patients with Chronic Pain

**DOI:** 10.1371/journal.pone.0154240

**Published:** 2016-04-27

**Authors:** B. Ojeda, A. Salazar, M. Dueñas, L. M. Torres, J. A. Mico, I. Failde

**Affiliations:** 1 Preventive Medicine and Public Health Area, The Observatory of Pain (External Chair of Pain), University of Cádiz, Cádiz, Spain; 2 Salus Infirmorum Faculty of Nursing, University of Cádiz, Cádiz, Spain; 3 Department of Anesthesiology-Critical Care and Pain Management, University Hospital “Puerta del Mar”, Cádiz, Spain; 4 Department of Neuroscience, Pharmacology and Psychiatry, CIBER of Mental Health, CIBERSAM, Instituto de Salud Carlos III, University of Cádiz, Cádiz, Spain; Banner Alzheimer's Institute, UNITED STATES

## Abstract

Patients with chronic pain often complain about cognitive difficulties, and since these symptoms represent an additional source of suffering and distress, evaluating the cognitive status of these patients with valid and reliable tests should be an important part of their overall assessment. Although cognitive impairment is a critical characteristic of pain, there is no specific measure designed to detect these effects in this population. The objective was to analyze the psychometric properties of the “Test Your Memory” (TYM) test in patients with chronic pain of three different origins. A cross-sectional study was carried out on 72 subjects free of pain and 254 patients suffering from different types of chronic pain: neuropathic pain (104), musculoskeletal pain (99) and fibromyalgia (51). The construct validity of the TYM was assessed using the Mini-Mental State Examination (MMSE), Hospital Anxiety and Depression Scale (HADs), Index-9 from MOS-sleep, SF-12, and through the intensity (Visual Analogical Scale) and duration of pain. An exploratory factor analysis was also performed and internal reliability was assessed using Cronbach’s alpha. After adjusting for potential confounders the TYM could distinguish between pain and pain-free patients, and it was correlated with the: MMSE (0.89, p<0.001); HAD-anxiety (-0.50, p<0.001) and HAD-depression scales (-0.52, p<0.001); MOS-sleep Index-9 (-0.49, p<0.001); and the physical (0.49, p < .001) and mental components (0.55, p < .001) of SF-12. The exploratory structure of the TYM showed an 8-factor solution that explained 53% of the variance, and Cronbach’s alpha was 0.66. The TYM is a valid and reliable screening instrument to assess cognitive function in chronic pain patients that will be of particular value in clinical situations.

## Introduction

Chronic pain (CP) has attracted significant attention in the last 30 years due to its prevalence and its impact on patients’ lives [1]. Indeed, the significant effect of CP on patients’ capacity to work and sleep, as well as on their everyday activities, means it has a predominant influence on their quality of life [2]. Since its definition by the International Association for the Study of Pain (IASP) in 1994: “*An unpleasant sensory and emotional experience associated with actual or potential tissue damage*, *or described in terms of such damage*”, the concept of pain has shifted from the passive to the active, assuming cognitive processes underlie pain perception. In fact, it is currently recognized that perceiving pain is the result of complex and dynamic interactions that both encode and process nociceptive signals, influencing normal cognitive function and placing additional demands on overall attentional resources [3,4].

When cognitive function has been assessed in CP patients, they appear to experience a mild impairment, most often reflecting poorer performance in memory tasks and executive functions [5–9]. However, interpreting the relationship between pain and cognitive function is complicated due to the variety of symptoms and circumstances that frequently affect these patients. Indeed, the relationship between pain and cognition is influenced by co-morbidities, such as depression, anxiety and sleep disorders [10], and by the consumption of analgesic medication, which has a deleterious effect on cognitive function [2]. Moreover, the specific cognitive changes associated with the distinct types of CP probably reflect the different mechanisms underlying these conditions, suggesting that diverse peripheral and central nervous system inputs have specific effects in the context of different types of pain [11]. Thus, the influence of pain on cognitive function is also likely to depend on the type of pain [6,7].

Nevertheless, when assessing this situation it is important to note that the scales used to assess cognitive functioning have not usually been specifically validated in CP patients. Moreover, those used are very broad, complex and time-consuming, sometimes requiring specialist health professionals to administer them [12], a challenge that must be overcome in order to implement them in clinical practice. The Mini Mental State Examination (MMSE) [13] is one of the most widely used scales for cognitive screening and it has also been applied to patients with CP [6,7,9,14,15]. However, this scale does not differentiate patterns in patients with mild cognitive impairments (MCI) [16] and it has not been validated in pain patients, raising questions about the validity of the results observed in these patients.

The Test Your Memory (TYM) scale was developed recently as a screening test to assess cognitive function in different groups of dementia patients. TYM proved to be useful to distinguish dementia from non-dementia cases, detecting almost all of the Alzheimer disease dementia cases with a cut-off ≤42/50 in the index paper [17]. It is short questionnaire that is easy and quick to apply and it has good internal consistency and has been shown to be more sensitive than the MMSE in detecting MCI [18–20].

Therefore, we set out to analyze the criterion, discriminant and structural validity, and internal reliability of this questionnaire in patients with CP of three different origins: neuropathic pain, musculoskeletal pain, and fibromyalgia.

## Methodology

### Subjects

This cross-sectional study included male and female patients between 18 and 60 years of age who attended the chronic pain unit at the university hospital in Cádiz (Spain). All the patients included had suffered chronic non-malignant pain for at least 3 months and they were divided into three separate groups in accordance with the cause and characteristics of the pain: musculoskeletal pain (MSK); neuropathic pain (NP); or fibromyalgia (FM). These groups were established according to the clinical examination carried out by pain specialists with extensive experience. MSK and NP cases were confirmed by objective tests such as electromyography, x-ray and advanced imaging, and were scored according to the ICD-10 criteria. In the case of FM, the American College of Rheumatology (ACR) 1990 classification criteria [21] were used for diagnosis. In addition, a group of adult patients (≥18 years old) that attended a primary health care consultation for preventive or minor health disorders and who were not diagnosed with acute or chronic pain, were included as a control group. The sample size estimation for the study was based on the objective of finding significant differences in the mean scores obtained by other studies in which cognitive function was assessed in similar groups of patients [6,15]. Based on the most conservative difference, given a 95% confidence level and an 80% power, we obtained the required sample sizes for each group. However, these sample sizes were slightly increased in anticipation of the use of non-parametric tests, since the assumption of normality was not reasonable in this case. Using the asymptotic relative efficiency (ARE) [22,23] in the worst possible scenario for the Mann-Whitney test (ARE = 0.864), the following sample sizes were obtained: NP = 99; FM = 99; MSK = 56; Control = 33.

All pain patients and control subjects were able to read and write, they were mentally and physically capable of participating in the study and they provided their written informed consent. Individuals already diagnosed with any type of dementia or those in the control group with an MMSE <24 or a score other than zero in the Visual Analog Scale were excluded from the study.

The study was conducted in accordance with the Helsinki Declaration, using Standard Working Procedures and Protocols, and it was approved by the Clinical Research Ethics Committee at the University Hospital “Puerta del Mar” in Cádiz, ensuring adherence to the norms of good clinical practice.

### Instruments and variables

Socio-demographic data and clinical variables (co-morbidities and cardiovascular risk factors like diabetes, hypercholesterolemia and/or hypertension) were recorded using a structured questionnaire and by consulting the patients’ clinical records. Information was collected on the pharmacological drugs being consumed by the patient at the time of the evaluation that might affect cognitive function, only recording whether the patient was taking certain drugs or not. The information regarding the specific effects of drugs on cognitive function was based on a list already drawn up using the technical data sheets of the products registered by the Spanish Agency for Medications and Healthcare Products. The intensity of pain experienced at the time of the evaluation was assessed using a Visual Analogue Scale (VAS), ranging from 0 (no pain) to 10 (the worst possible pain), and this was categorized as mild (<4), moderate (4–7) or severe (>7) pain (according to Collins et al., 1997) [24]. Information about the duration of the pain was also recorded and represented in quartiles.

The translated and adapted Spanish version of the TYM test [25] was used to assess cognitive function. The TYM is a double sided sheet that includes different tasks used to assess 10 cognitive domains: orientation, ability to copy a sentence, semantic knowledge, calculation, verbal fluency, similarities, naming, visuospatial abilities and recall of a copied sentence (see items and scores in Table 1). In addition, a final item assesses the ability to complete the test ranging from 1 (major help) to 5 (no help). The test is self-administered and takes around 5 minutes to complete, giving a maximum score of 50 points, where higher scores represent better cognitive function (all the instructions for TYM testing are available at www.tymtest.com). Importantly, it has been shown that the TYM is very sensitive and specific to detect cognitive impairment and dementia.

**Table 1:** Item content and TYM score.

TYM domains	No. of items	Score
1. Orientation (place & person orientation)	9	10
2. Copying	1	2
3. Retrograde memory (semantic knowledge)	2	3
4. Calculation	4	4
5. Verbal fluency	1	4
6. Similarities	2	4
7. Naming	5	5
8. Visuospatial ability 1 & 2 (clock)	2	7
9. Anterograde memory	1	6
10. Executive (ability to complete the test)	1	5
	28	**50**

The adapted and validated Spanish version of the Mini-Mental State Examination (MMSE) [26] was also used. The MMSE focuses on a range of cognitive functions that involve orientation, registration, attention and calculation, recall, language and visuoconstructional abilities. The test is scored from 0 to 30, where higher scores indicate better cognitive performance and a score above 24 reflects normal cognitive function.

The Medical Outcome Study (MOS) Sleep scale [27] was used to assess the quality and quantity of the patient’s sleep in the four weeks prior to evaluation. The questionnaire is an self-reported measure consisting of 12 items that generate six sub-scales and two summary indices that assess sleep quality (a 6 and 9 item index). This study only used the 9 item index, where the minimum score is 0 and the maximum score is 100 for the worst sleep quality. Likewise, as part of the MOS Sleep scale, the quantity of sleep was reported by patients as the average number of hours they slept each night, information that was subsequently categorized as optimal sleep (between 7 and 8 hours of sleep per night) and non-optimal sleep (less than 7 hours or more than 8 hours of sleep per night). This questionnaire has been validated in patients with pain and it displays good psychometric properties [28].

Depression and anxiety were evaluated using the 2 sub-scales of the Hospital Anxiety and Depression scale (HADs) [29]. The Spanish version of the scale has been shown to be appropriate, reliable and valid [30], and it is strongly recommended for use in patients with CP [31]. The two sub-scales each consist of 7 items scored as values from 0 to 21. A score above 10 in either of the sub-scales has been considered a clinically relevant optimal cut-off point to detect anxiety or depression in patients with CP [32].

The adapted Spanish version of the SF-12 v.1, by Alonso et al. [33], was also used to evaluate the patient’s health related quality of life (HRQL). This questionnaire has good psychometric properties and it uses 12 items, from which a summary of the physical and mental components (PCS and MCS) can be constructed. These components were calculated in accordance with the algorithms and the weightings established for the Spanish population, and they follow a standard T distribution, whereby the population scores present a mean of 50 and a standard deviation of 10. Higher scores in the summary components of the SF-12 (physical or mental) indicate a better state of health or HRQL.

All the information was collected on paper in the pain unit of the hospital by a previously-trained psychologist who was not the physician responsible for the clinical assessment of the patient. The study subjects performed the TYM test first, followed by the MMSE. Between the performances of both instruments, the administration of all the other instruments was performed. The testing procedure, in an isolated environment, lasted 1–1.5 h for each patient.

### Analysis

A descriptive analysis of the demographic and clinical characteristics of the subjects was performed (frequency, central tendency and dispersion measurements). In addition, the proportion of patients that obtained the lowest (floor) and the highest (ceiling) scores for the TYM and MMSE scales was calculated. The Kolmogorov-Smirnov test was used to determine the normality of the variables.

The homogeneity or internal consistency of the TYM was assessed using Cronbach’s alpha coefficient, and it was considered acceptable when alpha was ≥0.6 [34]. The correlation of each item with its own domain or dimension was also calculated and we assumed that items should be more closely related to their own dimension, correcting for overlap (item-internal consistency), than to the other dimensions (item-discriminant consistency).

To establish the structure of the TYM in the CP population, an Exploratory Factor Analysis (EFA) followed by Varimax rotation was performed. To determine the best solution for the number of factors, we first used the Kaiser rule, which involved dropping all components with eigenvalues <1 [35]. In addition, the scree plot was analyzed to determine how many components should be retained by plotting the eigenvalue against the factor number. The factor number is taken to be the point when the graph visually tends towards a horizontal line.

The literature often emphasizes that cognitive function in patients with chronic pain is affected by repeated factors. Thus, it appears that an altered state of anxiety or depression, or poor quality sleep leads to worse cognitive performance. In light of these results, we proposed to consider the cognitive function as a construct consisting of all those previously described associations. In other words, we assumed that in these patients with pain, certain circumstances will condition the results of the cognitive function scores. This assumption was reflected in different hypotheses that have been used to assess the validity of the "cognitive function" construct assessed through the TYM scale.

We established 14 hypotheses (H). Thus, given that CP can provoke emotional changes (anxiety and depression) and sleep alterations (quality and quantity), and given that these disorders are known to produce a deterioration in cognitive function in other patients [36–38], we hypothesized that the TYM score in the CP population should be inversely correlated with the scores of the HAD anxiety and depression subscales [5,39] (H1 and H2), with the score of index 9 of the MOS-sleep scale [40,41] (H3), and with the duration (H4) and intensity (H5) of pain [42]. Moreover, we postulated that the PCS and MCS scores of the SF-12 scale should be positively correlated with the TYM scores (H6 and H7). As such, the Spearman correlation coefficient was calculated for these parameters.

The evaluation of the construct validity also included an analysis of the discriminant validity in defined subgroups of patients, hypothesizing that lower TYM scores would be obtained from patients with CP compared to the control group [15] (H8). Similarly, it was postulated that the TYM score would differ between the three groups of CP patients (FM, NP and MSK) (H9) given that the characteristics of each type of pain may affect cognitive function distinctly [7,43]. Likewise, we considered that the patients who fulfilled their sleep needs (optimal sleep: between 7 and 8 hours per day [27]) would have higher TYM scores than the patients who slept less than 7 hours or more than 8 hours per day (H10). In addition, the patients with anxiety or depression (HAD-A and/or HAD-D scores >10) should have lower TYM scores than the patients free of these disorders [9] (H11 and H12). Finally, we also assumed that patients with more severe (>7 in VAS) (H13) and longer lasting pain (last quartile) (H14) would have a lower TYM score [2]. Such comparisons were made with the Kruskal-Wallis H and Mann-Whitney U test in accordance with the variables analyzed.

The MMSE reference questionnaire was used to evaluate criterion validity, considering that this scale should be closely related to the TYM score, a hypothesis tested with the Spearman’s rank correlation test.

To analyze each hypothesis and eliminate the potential confounders related to differences in sex, age, academic level, anxiety and depression scores, quality of sleep, treatment with drugs that potentially affect cognitive function, and cardiovascular risk factors between the case and control and between the tree groups of CP patients, the scores of TYM and MMSE were adjusted by these variables prior to the comparisons.

All the analyses were performed with the SPSS statistical package (Ver. 21).

## Results

### Population characteristics

A total of 336 subjects were recruited onto this study from November 2010 to April 2012. Of the individuals approached, six were excluded as they reported no pain at the time of the evaluation (VAS = 0), three refused to participate and one could not complete the test due to visual impairment. Thus, a final cohort of 254 subjects suffering CP (51 subjects with FM, 104 with NP and 99 with MSK: Table 2) and 72 pain-free controls were studied. The average age was slightly higher in patients with CP, as was the percentage of women (particularly among the patients with FM: Table 3). There were also differences in the level of studies completed that could have affected the scoring in the cognitive function tests, CP patients generally having a lower level of education. Likewise, CP patients had a greater tendency towards cardiovascular risk factors, although the difference with the control group and between the three CP groups was not significant (Table 3). By contrast, there was a significant four-fold increase in the frequency of drug consumption that potentially deteriorated cognitive function in subjects with pain (83.9% vs 20.8%, p<0.001), especially in the NP group (Table 3). The CP group obtained higher scores in the scales of anxiety and depression, as well as in the sleep index, reflected by a higher proportion of individuals with non-optimal sleep compared to the control group. Likewise, patients with CP performed worse in both the physical and the mental components of the SF-12 (Table 3).

**Table 2:** Frequencies of the causes underlying neuropathic or musculoskeletal pain.

Classification and frequencies of diseases (ICD-10) N = 254	
NP (n = 104)	%
- Disc disease	42.3
- Postlaminectomy syndrome	28.8
- Complex regional pain syndrome I (CRPS I)	7.7
- Trigeminal neuralgia	5.8
- Neuroma	4.8
- Diabetic neuropathy	2.9
- Complex regional pain syndrome II (CRPS II)	2.9
- Post-traumatic neuralgia	2.9
- Central pain syndrome	1
- Carpal tunnel syndrome	1
**MSK (n = 99)**	**%**
- Disc disease	46.5
- Osteoarthritis (Arthrosis)	25.3
- Postlaminectomy syndrome	16.2
- Muscle disorders	9.1
- Rheumatoid arthritis	3.0

**Table 3:** Demographic and clinical characteristics of the chronic pain patients and the reference group.

	Cases (n = 254)	Controls (n = 72)	p	NP (n = 104)	MSK (n = 99)	FM (n = 51)	p
**Sex** female (%)	63.8	56.9	.179^a^	52.9	58.6	96.1	< .001^a^
**Age in years** Mean (SD)	47.4 (8.8)	40.0 (11.1)	< .001^b^	45.6 (8.7)	47.6 (9.4)	50.8 (6.7)	.002^c^
**Academic level** (%)			< .001^a^				.271^a^
No education	11.8	5.6		10.6	14.1	9.8	
Primary education	39.0	22.2		47.1	34.3	31.4	
Secondary/Vocational training	40.9	36.1		37.5	40.4	49.0	
University studies	8.3	36.1		4.8	11.1	9.8	
**Cardiovascular risk factors** (%)							
Diabetes	10.6	8.3	.568^a^	11.5	10.1	9.8	.925^a^
Hypercholesterolemia	16.1	8.3	.096^a^	11.5	17.2	23.5	.152^a^
Hypertension	16.1	18.1	.700^a^	13.5	19.2	15.7	.538^a^
**Pharmacological drugs** (%Yes)	83.9	20.8	< .001^a^	89.4	75.8	88.2	.019^a^
**Antidepressants**	36.2	5.6	< .001^a^	33.7	27.3	58.8	.001^a^
**BZD**	42.1	8.3	< .001^a^	39.4	39.4	52.9	.216^a^
**Anticonvulsants**	45.7	1.4	< .001^a^	52.9	44.4	33.3	.068^a^
**Opioids**	53.9	0	< .001^a^	61.5	52.5	41.2	.054^a^
**NSAID**	8.3	2.8	.082^a^	7.7	10.1	5.9	.648^a^
**HAD-D** Mean(SD)	7.9 (5.2)	2.1 (2.8)	< .001^b^	7.9 (5.3)	7.3 (5.3)	8.8 (4.6)	.149^c^
**HAD-A** Mean(SD)	9.4 (4.9)	3.6 (4.0)	< .001^b^	9.0 (4.8)	8.9 (5.0)	11.4 (4.6)	.004^c^
**MCS** Mean(SD)	35.1 (12.6)	49.0 (12.4)	< .001^b^	36.1 (12.4)	36.3 (12.9)	30.7 (11.7)	.020^c^
**PCS** Mean(SD)	33.1 (8.4)	54.1 (4.8)	< .001^b^	32.0 (8.2)	34.4 (8.8)	32.9 (8.0)	.201^c^
**I9** Mean(SD)	52.4 (21.7)	29.3 (13.2)	< .001^b^	52.4 (21.8)	49.4 (22.4)	58.2 (19.1)	.072^c^
**NO-optimal sleep** (%)	80.3	47.2	< .001^a^	81.7	75.8	86.3	.276^a^
**Intensity (0–10)** Mean (SD)	6.6 (1.9)	-	-	6.6 (1.85)	6.7 (2.0)	6.6 (1.7)	.860^c^
Mild (<4) %	7.1	-	-	5.8	9.1	5.9	.489^a^
Moderate (4–7) %	59.4	-		63.5	52.5	64.7	
Severe (>7) %	33.5	-		30.8	38.4	29.4	
**Duration in months** Mean (SD)	108.5 (97.6)	-	-	92.1 (82.7)	102.0 (97.4)	154.5 (112.7)	.001^c^

Statistical tests

^a^ Chi-Square

^b^ U-Mann Whitney

cH-Kruskall Wallis.

The average VAS score was very similar for all three groups of CP patients analyzed, although when this variable was categorized the proportion of patients with severe pain was higher among the MSK patients (38.4% VAS>7: Table 3). In terms of duration, the patients with FM had suffered pain for more than 12 years, longer than in the other two groups (Table 3). While this was reflected by higher average scores for anxiety in the FM group, the HAD scores for depression were very similar in all three groups (Table 3). In terms of the physical and mental components of the SF-12, patients with NP had the lowest PCS value, while those with FM achieved the lowest MCS scores (Table 3).

### Descriptive analyses of TYM and MMSE

When considering the mean TYM and MMSE scores (Table 4), the scores from both instruments were lower for the subjects with pain than for the pain-free controls (p<0.005). The effect size, measured by Cohen’s d [44], is medium (d = 0.65) for the mean TYM scores, and large (d = 1.08) for the mean MMSE scores. On the other hand, the means were similar in the 3 groups of patients with pain. A stronger ceiling effect was observed in the MMSE than in the TYM, and no missing or unanswered items were registered with either instrument.

**Table 4:** Distribution of the crude scores for the TYM and MMSE scales.

		Cases (254)	Controls (72)	Cohen’s d	NP (104)	MSK (99)	FM (51)
**TYM**	Mean (SD)	40.5 (5.5)	43.9 (4.1)	0.65	40.5 (6.2)	40.7 (5.4)	40.2 (4.4)
	IC (95%)	39.8–41.2	31		39.3–41.7	39.6–41.8	38.9–41.45
	Minimum	21	31		21	26	31
	Maximum	50	50		50	49	50
	Floor (%)	0.4	1.4		1	3	2
	Ceiling (%)	1.2	5.6		1.9	1	2
**MMSE**	Mean (SD)	26.9 (2.2)	29.1 (1.4)	1.08	26.9 (2.2)	27 (2.3)	26.8 (2.3)
	IC (95%)	26.7–27.2	28.8–29.4		26.5–27.4	26.5–27.4	26.2–27.5
	Minimum	17	24		17	20	19
	Maximum	30	30		30	30	30
	Floor (%)	0.4	1.4		1	1	2
	Ceiling (%)	9.4	58.3		8.7	11.1	7.8

### Reliability of the TYM

The internal consistency of the TYM in CP patients assessed with Cronbach’s alpha was 0.66 for the complete scale. Moreover, all the items in the scale showed a stronger correlation with the theoretical domains (item-internal consistency) than with the other domains (item-discriminant consistency: Table 5).

**Table 5:** Internal consistency and homogeneity of the TYM scale in the population studied (n = 254).

		Range of item correlations	
Domains	No. of items	Item-internal consistency^a^	Item-discriminant consistency^b^
Orientation	9	.20-.50	.00-.31
Semantic knowledge	2	.76-.77	.06-.21
Calculation	4	.51-.77	.02-.19
Similarities	2	.72-.80	.03-.16
Naming	5	.12-.74	.00-.11
Visuospatial	2	.76-.76	.04-.16

^a^ Minimum-maximum correlation of the item with its own scale.

^b^ Minimum-maximum correlation of the items with other scales.

### Structural validity

**Fig 1:**
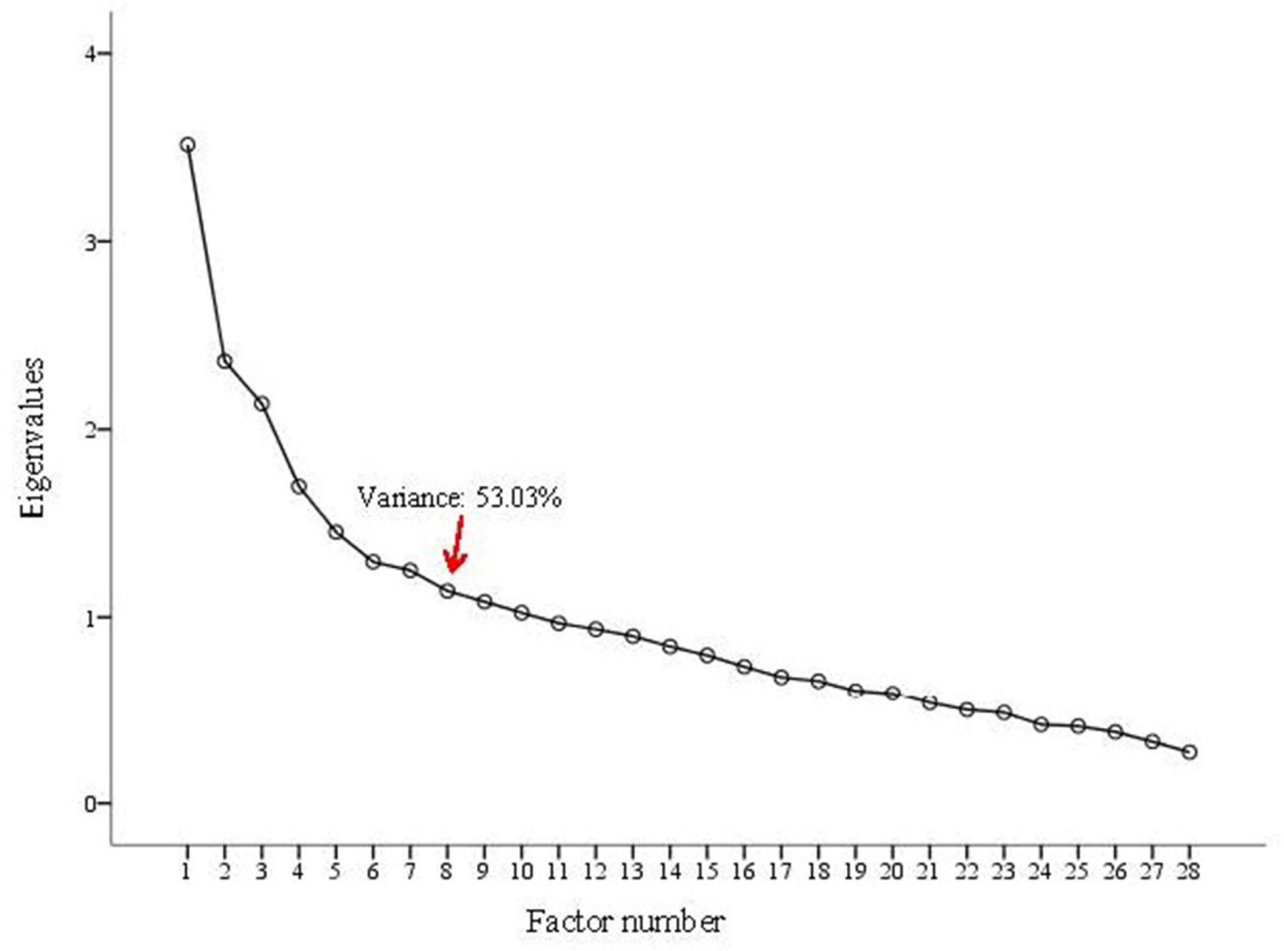
TYM Scree plot (n = 254).

The Exploratory Factor Analysis (EFA) performed on the TYM extracted 10 factors with eigenvalues >1 (Kaiser’s criterion), which explained the variability of 60.5%. However, according to the scree plot, from the eighth factor onwards the graph tended towards the horizontal (Fig 1). Therefore, we performed the EFA to extract 8 factors and we found that the items included in the dimensions Naming, Calculation and Visuospatial ability of the original TYM structure coincided precisely with factors 2, 4 and 7 of the EFA. The items in the Verbal Fluency and Similarities dimensions were unified in factor 6, while factors 1, 3 and 8 presented stronger crossloading, especially those that formed part of the Orientation dimension in the original scale (S1 Table). This structure explained 53.3% of the variance.

### Construct validity

Analyzing the validity of the construct, significant correlations were found between the adjusted TYM scores and the anxiety (-0.52; p<0.001) or depression HAD scales (-0.50; p<0.001), as well as with the MCS (0.49; p<0.001) and PCS (0.55; p<0.001) of the SF-12, and with index 9 in the MOS scale (-0.49; p<0.001). There was also a significant negative correlation between the TYM score and pain intensity (-0.13; p<0.005), although no correlation was observed between TYM and the duration of pain (-0.03; p = 0.680).

**Fig 2:**
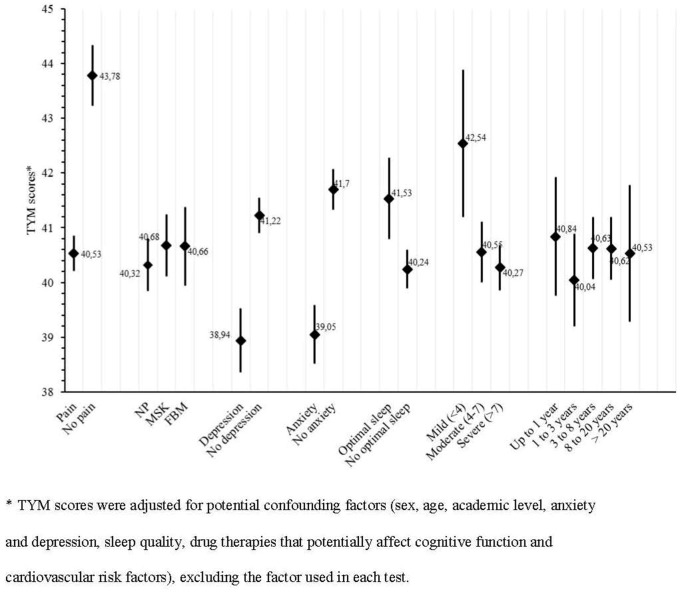
Discriminant validity—TYM adjusted mean scores according to different categories* (n = 254).

Analyzing the discriminant validity, patients with CP obtained lower adjusted average TYM scores than the control subjects (individuals without pain), although no differences were found in the scores between the three groups of patients who suffered pain of different origins (Fig 2). Conversely, patients that recorded optimal sleep obtained higher adjusted scores in the TYM than the patients that recorded non-optimal sleep (Fig 2), and those patients with anxiety and/or depression obtained lower adjusted scores than patients without these co-morbidities. When considering the intensity of pain in each of the three categories (mild/moderate/severe), the lowest adjusted scores in the TYM were associated with more severe pain. However, no differences were found between the quartiles in terms of the duration of pain (Fig 2). Finally, when the validity criterion was analyzed the TYM adjusted score was strongly correlated with the adjusted scores of the MMSE (r = 0.89; p<0.001).

## Discussion

This study demonstrates that the TYM is an adequate screening tool to explore cognitive function in CP patients, confirming the valuable psychometric properties of the instrument. Indeed, this represents the first study to analyze the structure of this TYM scale.

The results supported 11 of the 14 hypotheses proposed as constructs in the validation process, including the assumption that significant differences between cases and controls would exist in cognitive performance. They showed a narrow difference between the groups, demonstrating, as expected, that the impairment of cognitive function most frequently reported in patients with CP is mild, and therefore the difference with subjects without pain should not be too large. Studies that have previously used screening measures such as the Mini Mental State Exam (MMSE) [45,46], or the Cognitive Capacity Screening Exam (CCSE) [14] found that CP patients performed poorly compared with controls. Furthermore, in other studies the prevalence of clinically relevant cognitive impairment (measured as an MMSE score <24) was higher in chronic pain patients in comparison with the general population [6,7]. However, these studies did not take into account factors with a potentially negative affect on cognitive function, so the differences found might in fact be greater. In accordance with a previously published paper carried out in other types of chronic pain patients [47,48], this study was not suitable to distinguish the cognitive differences between the distinct types of pain, and failed to confirm that the type of clinical pain experienced by patients depends on the type of nociceptive stimulus (i.e.: inflammation or neuropathic) and that, in turn, such nociceptive stimuli trigger different nociceptive mechanisms that may affect cognitive function distinctly [6,7]. One explanation for this result could be the inaccuracy of the diagnosis of the patients as a result of the poorly-defined boundaries between diagnoses of neuropathic and musculoskeletal pain, which might have been a key reason for the absence of some significant differences. However, the expertise level of the physician assessing the patients in the study makes this quite unlikely. Another potential explanation could be the insensitivity of the test. Nevertheless, more specific studies will be needed to measure the sensitivity of the TYM and also to understand whether and how the type of pain interferes with cognitive function in these patients.

The two-way relationship between pain and affective disturbances has been largely addressed in the literature and multiple explanations have been proposed to justify these different pathways [49]. However, this topic has not been resolved despite the fact that the comorbidity of affective disorder in CP is important from a clinical and research standpoint. In this study, anxiety and depression were more frequent in the CP patients than the control groups (HADs-Anxiety (≥10): 43.3% vs. 6.9% and HADs-Depression (≥10): 29.1% vs. 0%), and the degree of anxiety and/or depression was associated with impaired cognitive function defined by the TYM test, confirming the hypothesis of the relationship between the affective disorder and cognitive function in patients with CP. The negative influence of anxiety and/or depression on cognitive function has been widely assessed, not just in pain but also in patients with other pathologies [50]. Accordingly, a reduction in brain volume has been seen in patients who suffer strong depression or chronic stress, changes that would appear to contribute to emotional and cognitive impairment [51]. However, cognitive impairment could be a possible antecedent of emotional disturbance such as depression and anxiety in CP patients.

Separating pain-related effects on cognition from the effects of emotional distress is one of the main difficulties in studying pain-cognition interactions. Most pain patients suffer from depression, anxiety, stress, anger or a combination of these and related factors, all of which affect cognitive functioning directly or indirectly, via apathy, medication effects, fatigue, and disordered sleep [52]. Notably, several studies that quantitated cognitive impairment found that such factors fully accounted for the observed numerical decrements [53]. Yet even in such studies, it is possible that cognitive impairments were initially the result of pain and only subsequently became statistically correlated with indices of depression. In many cases the cognitive deficits may be at least partially explained by patients’ depression and emotional distress. While these mood factors may account for cognitive difficulties, other explanations are possible, some of which have not yet been evaluated in depth.

Pain intensity was correlated with the TYM test score, with lower scores among patients with severe pain. Similar findings were established in a mixed population of pain patients, where those who reported high intensity pain performed worse in a demanding attentional task than subjects with low intensity pain [42]. This was explained by more intense painful sensations placing a greater demand on attention, leaving fewer attentional resources to be employed in other cognitive tasks that might be performed concurrently. Despite focusing on distinct tasks to those used in this mixed population, our data might be consistent with this attentional theory given that performing the TYM test demands certain attention and the effect of distraction due to pain could also be prejudicial. Thus, it would be interesting to study the sensitivity to changes in the TYM test, and to assess whether cognitive dysfunction is reversible and dependent on treatment outcomes, as suggested previously [54].

There is a wealth of evidence that sleep-dependent mechanisms affect the neural plasticity leading to the consolidation of learning and memory [55]. This interaction has been analyzed in FM [40] and in mixed pain [56], with sleep disturbances apparently having a negative influence on cognition. These results are consistent with the data presented here, where the lower scores obtained in the TYM scale were associated with non-optimal sleep patterns. Indeed, there was an explicit correlation between poorer cognitive functioning and worse sleep quality, which suggests that sleep quality may influence the relationship between pain and cognitive impairment [9,41].

Our study demonstrates a relationship between poorer cognitive performance and a worsening in the mental and physical HRQL (Health Related Quality of Life) components. Prolonged pain negatively affects mental health and the ability to perform everyday tasks, which translates into a poorer perception of the patient's physical and mental status [57]. However, this relationship is difficult to establish in this study, as the changes in HRQL could be a cause and a consequence of the CP patient's cognitive state. Nevertheless, HRQL is increasingly being recognized as one of the most important parameters to be measured in the evaluation of medical therapies, including those for pain management.

In relation to the structure of the TYM for pain patients, the 8 factor structure was still the most relevant due to the distribution of the items for the different factors and despite the loss of variance that could be explained. It is significant that 3 of the 8 factors described in the EFA matched the naming, calculation and visuospatial ability domains of the original scale [17]. Moreover, the similarities and verbal fluency domains were grouped in the same factor, given that they share language as a cognitive trait [58]. However, the remaining items were distributed following a slightly different pattern than the original version and they showed relevant crossloading, indicating that further studies will be needed to analyze the structure of the TYM.

Finally, it should be noted that the TYM is a self-administered test that is easy and quick to use (administered to pain subjects in 10 min on average), and it has more features than the MMSE. One is the multi-task feature that opens the test to greater variability in terms of the results and the exploration of more cognitive aspects than with the MMSE test. Another advantage of the TYM is that the ceiling effect is weaker than with the MMSE. These features are particularly relevant given that the cognitive impairment observed in CP populations is generally milder than in other populations studied.

### Strengths and weaknesses of the study

One strength of this study is that it used rigorous diagnostic criteria to analyze 3 distinct groups of patients with CP. Likewise, the tools with which the TYM was compared, including the HAD, MOS-sleep and SF-12, have psychometric properties appropriate to patients with pain. Moreover, the TYM scores were adjusted to many variables common to CP patients with known effects on cognitive performance, including educational level, drug consumption and cardiovascular risk factors, which were taken into account in the validation process. This study demonstrates the usefulness of an instrument that overcomes the limitations that have become evident in the different studies where patients with CP have been assessed with tests that have not been validated with such patients, which may result in lower statistical power and potential bias. Considering these variables, a more accurate estimation of the test results was performed.

In terms of the possible limitations of the study, no cut-off point has been established for the scale, although an analysis using cut-off values was considered somewhat dubious due to the paucity of studies in patients with CP [9]. Nonetheless, adequate diagnostic criteria to establish the degree of cognitive impairment in the population were not employed in this study. This limitation should be corrected in future research aimed at validating the TYM test in patients with pain. Thus, further longitudinal research will be needed to add consistency to causal conclusions and in order to identify the minimal differences clinically important differences in the TYM score of CP patients.

## Conclusion

This is the first study to assess the psychometric properties of the TYM in patients with CP of 3 different origins. The results obtained show that the TYM is a valid and reliable tool to screen the mild deterioration or decline in cognitive function frequently reported in patients with CP. When coupled to its ease of use, the TYM test is clearly an interesting alternative to apply to CP patients in regular clinical practice.

## Supporting Information

S1 Data Data Raw for PlosOne. (XLSX)

S1 Table Exploratory Factor Analysis of the TYM (n = 254). (DOC)
