# Supplementary material for: Assessing the Construct Validity and Internal Reliability of the Screening Tool Test Your Memory in Patients with Chronic Pain
Source: PLoS One. 2016 Apr 27;11(4):e0154240. doi: 10.1371/journal.pone.0154240 (PMC4847905; doi:10.1371/journal.pone.0154240)
Supplement: S1 Table — (DOC) [file pone.0154240.s002.doc]

**Table Supplemental Digital Content 1.** Exploratory Factor Analysis of the TYM (n=254).

|  | 1 | 2 | 3 | 4 | 5 | 6 | 7 | 8 |
| --- | --- | --- | --- | --- | --- | --- | --- | --- |
| Orientation  TYM 1.1  TYM 1.2  TYM 1.3  TYM 1.4  TYM 1.5  TYM 1.6  TYM 1.7  TYM 1.8  TYM 1.9 | -,069  -,181  -,077  -,070  ,268  -,064  **,809**  **,805**  **,782** | ,149  -,014  -,011  ,097  ,000  -,063  ,052  -,003  ,007 | ,131  **,530**  -,138  ,228  -,006  ,065  ,040  ,124  ,080 | -,225  -,030  ,107  ,097  -,048  -,022  -,010  ,032  ,066 | -,174  ,080  **,429**  **,658**  **,634**  **,728**  ,078  -,048  -,046 | -,117  ,251  ,013  ,057  ,230  -,118  ,079  -,061  ,060 | ,028  -,297  -,024  ,001  ,188  -,049  -,013  ,123  -,086 | **,399**  -,093  ,477  ,040  -,080  ,069  -,052  -,074  ,091 |
| Copying  TYM 2 | ,360 | -,049 | ,**469** | ,078 | ,035 | -,080 | ,132 | ,002 |
| Semantic knowledge  TYM 3.1  TYM 3.2 | ,051  ,041 | -,166  ,064 | ,488  ,123 | -,099  ,172 | -,031  ,138 | ,076  ,206 | ,069  -,015 | **,387**  **,703** |
| Calculation  TYM 4.1  TYM 4.2  TYM 4.3  TYM 4.4 | -,105  ,069  ,184  ,022 | -,075  ,011  ,027  -,001 | -,088  ,184  ,373  -,022 | **,760**  **,696**  **,593**  **,606** | ,027  ,040  ,049  -,014 | ,115  ,098  ,004  ,004 | ,177  -,207  ,050  ,129 | ,082  -,126  ,014  ,066 |
| Verbal fluency  TYM 5 | ,146 | -,110 | -,081 | ,124 | ,140 | **,700** | ,054 | ,124 |
| Similarities  TYM 6.1  TYM 6.2 | ,024  -,201 | ,149  -,008 | ,270  ,014 | -,012  ,184 | -,006  -,114 | **,640**  **,491** | -,126  ,355 | ,046  -,049 |
| Naming  TYM 7.1  TYM 7.2  TYM 7.3  TYM 7.4  TYM 7.5 | -,013  ,127  -,064  -,052  ,044 | **,570**  **,640**  **,797**  **,650**  **,656** | -,033  -,054  -,001  ,019  -,005 | -,020  ,039  ,012  ,057  -,154 | -,052  ,056  ,039  -,081  ,036 | -,180  ,171  -,044  ,058  ,014 | -,051  -,028  ,098  -,260  ,226 | ,198  -,026  ,041  -,159  ,047 |
| Visuospatial ability  TYM 8.1  TYM 8.2 | ,020  ,049 | ,011  ,022 | ,084  ,299 | ,093  ,050 | -,017  ,159 | -,044  ,160 | **,667**  **,601** | ,103  -,210 |
| Anterograde memory  TYM 9 | ,067 | ,025 | **,640** | ,090 | ,059 | ,147 | ,217 | ,197 |
| Executive  TYM 10 | ,245 | ,001 | **,697** | ,212 | ,188 | -,123 | ,187 | -,051 |
| Eigenvalues | 3.51 | 2.36 | 2.14 | 1.7 | 1.45 | 1.29 | 1.25 | 1.14 |
